# Patient perspectives on epidermal growth factor receptor inhibitor-induced skin toxicities: a qualitative study

**DOI:** 10.3389/fpsyg.2025.1731646

**Published:** 2026-01-20

**Authors:** Kexuan Li, Youfen Fan, Qionghui Sun

**Affiliations:** Burn Department, Ningbo No.2 Hospital, Ningbo, Zhejiang, China

**Keywords:** cancer, epidermal growth factor receptor inhibitors, qualitative study, skin toxicity symptoms, target therapy

## Abstract

**Objective:**

This study systematically explored the experience and supportive care needs of patients with cancer developing skin toxicity following Epidermal Growth Factor Receptor Inhibitor (EGFRI) treatment.

**Methods:**

A descriptive qualitative study was conducted. A total of 9 patients with cancer recruited from a single tertiary hospital in Zhejiang Province between July and August 2024 participated in semi-structured interviews. Data were analyzed using Colaizzi’s seven-step method to systematically derive themes related to patients’ experiences and care needs.

**Results:**

This study extracted a total of five themes and thirteen sub-themes, which included adverse physiological experiences, coexistence of negative and positive emotional experiences, impacts on daily life, diverse coping styles, and desire for medical support and help.

**Conclusion:**

Clinical staff should prioritize attention to the physical and psychological distress experienced by patients with cancer with EGFRI-induced skin toxicities. Implementing enhanced symptom evaluation and management, providing tailored psychological support, and offering professional guidance can help patients effectively cope with their symptoms and foster their self-growth.

## 1 Introduction

Cancer remains a leading cause of death globally, significantly affecting human longevity. Projections indicate a substantial rise in incidence, with new cancer cases expected to reach approximately 28.4 million worldwide by 2040, marking a 47% increase from the 19.29 million cases recorded in 2020 (Sung et al., 2021). This trajectory confirms that cancer is a major global public health concern. Targeted drug therapy is an important anticancer strategy. Compared to conventional treatments such as chemoradiotherapy, targeted drugs exhibit high selectivity for tumor cells by acting on specific binding sites, resulting in less damage to normal cells and generally better patient tolerance (Gong, 2017). However, while effectively inhibiting tumor progression, EGFRIs frequently disrupt the structural homeostasis of the skin and its appendages, leading to damage to normal skin tissues (Pinto et al., 2016). EGFRI-induced skin toxicity is the most prevalent adverse effect of this class of drugs. These dermatological adverse events not only impact the patient’s physical appearance but also significantly compromise their psychological well-being and quality of life (Huang and Ma, 2018). Critically, intolerable skin toxicities can lead to adverse clinical outcomes, including treatment delays, dose modifications, and discontinuation (Wang et al., 2017). Therefore, the management and prevention of EGFRI-induced skin toxicity are crucial areas of focus in supportive oncology research. Currently, existing studies are anchored in a clinician-observed, grading-focused paradigm. This approach prioritizes the objective assessment of physical signs (e.g., using CTCAE criteria) and reactive biomedical interventions (Zhang et al., 2019; Lacouture et al., 2021). However, this paradigm inherently captures only the exterior manifestation of toxicity, failing to encompass a patient’s interior reality. Qualitative research (QR) is particularly well suited to explore this dimension (Li et al., 2025). As a methodology designed to investigate complex human experiences and the meanings individuals assign to them within natural contexts, QR provides the necessary tools for an in-depth, systematic examination of subjective patient experiences that quantitative measures alone cannot reveal (Wang, 2022)^.^ Consequently, the in-depth, patient-centered insights that qualitative research can generate represent a key opportunity and have not yet been fully captured in the context of this specific EGFRI-induced skin toxicity. This study employed a qualitative design to systematically analyze the symptom experience and supportive care needs of patients with cancer with EGFRI-induced skin toxicities directly from their perspective, which aims to provide a reference for clinical practice.

## 2 Materials and methods

### 2.1 Interviewee recruitment

To ensure the depth, completeness, and richness of the data, a purposive sampling strategy incorporating the principle of maximum variation was employed. This approach aimed to recruit a diverse group of patients with cancer with EGFRI-induced skin toxicities, enabling a comprehensive understanding of the experience across different backgrounds, while ensuring that common characteristics were extracted. Patients with cancer who met the inclusion criteria were recruited from the Department of Medical Oncology at a tertiary care hospital in Zhejiang Province between July 2024 and August 2024.

The inclusion criteria for interviewees were (1) pathologically or cytologically confirmed malignant tumor; (2) age ≥ 18 years; (3) expected survival > 3 months; (4) positive reaction for EGFR gene mutation; (5) use of EGFRIs such as gefitinib, erlotinib, icotinib, and cetuximab; (6) rash or any other adverse skin reactions (including skin itching, dryness, fissures, and desquamation); (7) skin lesions graded according to the specific criteria for EGFRI-induced skin lesions established in 2007 in China (Table 1); and (8) adequate expression and communication ability and voluntary participation in the interview.

**Table 1:** Chinese grading of the extent of EGFRIs in 2007.

Grade	Criteria
I	The extent of the rash was limited (mainly confined to the head, face, and upper trunk), with few subjective symptoms, no impact on daily life, and no secondary infection.
II	It has a wide range of mild subjective symptoms, a slight impact on daily life, and no signs of secondary infection.
III	It has a wide range of severe subjective symptoms, a great impact on daily life, and the possibility of secondary infection.

The exclusion criteria for interviewees were as follows: (1) diagnosed with mental illness with severe cognitive impairment that would prevent cooperation; (2) critically ill with organ dysfunction (e.g., heart, liver, and kidney); (3) currently receiving chemoradiotherapy or other treatment methods besides EGFRIs; and (4) combined with other skin diseases such as psoriasis, atopic dermatitis, and eczema.

The sample size was guided by the principle of data saturation, defined as the point at which no new themes or sub-themes emerged from the data analysis, indicating information redundancy. After analyzing the 9th transcript, two consecutive interviews yielded no new meaning units or thematic dimensions. Upon collective deliberation by the research team, thematic saturation was determined to have been reached. Therefore, data from the first nine participants were included in the final analysis. The demographic and clinical data of the interviewees are presented in Table 2.

**Table 2:** General characteristics of included interviewees.

Number	Gender	Age	Education level	Residence	Occupation	Cancer	Targeted agents	Skin toxicity symptom grade
P1	Female	64	Junior high school	Rural	Retired	Rectal Cancer	Cetuximab	III
P2	Male	67	Junior high school	Urban	Retired	Rectal Cancer	Cetuximab	III
P3	Male	21	Undergraduate	Urban	Student	Colon Cancer	Cetuximab	I
P4	Male	47	Junior high school	Rural	Worker	Lung Cancer	Erlotinib	III
P5	Male	65	High school	Rural	Farmer	Colon Cancer	Cetuximab	III
P6	Female	56	Undergraduate	Urban	Clerk	Lung Cancer	Osimertinib	III
P7	Male	55	Junior high school	Rural	Clerk	Rectal Cancer	Panitumumab	II
P8	Male	74	Junior high school	Urban	Retired	Lung Cancer	Erlotinib	II
P9	Female	59	Junior high school	Urban	Clerk	Lung Cancer	Osimertinib	III

P denotes patient.

### 2.2 Interview guideline design

This study adopted a descriptive phenomenological approach utilizing semi-structured interviews to gain an in-depth understanding of the lived experiences of patients with cancer with EGFRI-induced skin toxicity. To ensure the rigor and content validity of the interview process, the development of the interview guide followed a systematic, multi-stage process. First, a preliminary thinking frame was constructed based on a comprehensive review of the scientific literature on the pathophysiology, clinical manifestations, and reported patient experiences with EGFRI-related skin toxicity. This review identified key domains that are essential to explore, including the sensory perception of symptoms, emotional and psychosocial impact, and self-management behaviors. Subsequently, the draft guide was refined through iterative discussions within our multidisciplinary research team, which included qualitative methodologies and clinical oncologists. These discussions focused on ensuring that the questions were non-leading and comprehensively covered the phenomenological dimensions of the lived experience. To establish expert validity, the revised guide was reviewed by an external panel comprising a senior qualitative research methodologist and two oncology clinical nurse specialists with extensive experience in managing targeted therapy side effects. Their feedback primarily pertained to the clarity, sequencing, and cultural appropriateness of the probing questions. Consensus suggestions were incorporated, resulting in a final set of core questions. The core questions included the following: (1) Do you have any discomfort with your skin during the use of targeted drugs such as EGFRIs? What symptoms and manifestations do you experience on your skin? What are your feelings and experiences? How do you perceive these symptoms? (2) Can you describe the process of the occurrence of these symptoms? (3) What are the effects of these symptoms on your daily life such as socialization, work, mood, etc.? (4) When coping with the above skin problems, do you encounter difficulties, and how do you adjust and cope? What do you think are the ways to improve these skin symptoms? How do you know? (5) What professional guidance and assistance do you want to provide to relieve these symptoms?

### 2.3 Data collection

Prior to the interview, the researcher confirmed a convenient time and place for each interviewee. The researcher chose a quiet and comfortable location such as the demonstration classroom or an empty ward. Before starting, the researcher explained the purpose and significance of the study, obtained written informed consent, and requested permission for synchronous recording. During the interviews, a flexible approach was adopted, allowing the researcher to adjust the order and mode of the questions based on participants’ responses to ensure a flowing conversation. Non-verbal cues, such as expressions, postures, and movements, were also carefully observed and noted to capture comprehensive contextual information. Throughout the interview, the researcher maintained a neutral stance, avoiding leading questions or suggestions and refraining from interrupting the interviewee. The researcher actively listened, observed, and employed techniques, such as questioning, listening, responding, repetition, clarification, summary, and other methods and skills, to ensure data richness and accuracy. Each one-on-one in-depth interview lasted 30–60 min.

### 2.4 Data analyses

This study rigorously adhered to Colaizzi’s (1978) seven-step phenomenological analysis method. All analyses were performed manually on interview transcripts without the use of qualitative data analysis software. To ensure the trustworthiness of the study, the process incorporated investigator triangulation, reflexive practices, and member checks.

The specific operational procedure was as follows: (1) Familiarization: Interviews were transcribed verbatim by the primary researcher within 24 h. The research team immersed themselves in the data through repeated reading of the texts and listening to audio recordings. (2) Identifying Significant Statements: Two researchers trained in qualitative methods independently reviewed all transcripts, highlighting all statements pertaining to the experience of skin toxicity. (3) Formulating Meanings: Subsequently, two researchers held consensus meetings to discuss each independently highlighted statement. They extracted the phenomenological essence to formulate “meaning units.” For example, the raw statement “It seems like the targeted drug is helping my body detoxify, and what’s coming out are toxic rashes” was formulated as “Reinterpreting physical symptoms as a positive signal of treatment efficacy.” This consensus framework guided all subsequent analyses. (4) Clustering into Themes: All consensual meaning units were extracted, physically compared, sorted, and clustered. Through multiple team meetings, units expressing similar core ideas were grouped to form preliminary sub-themes, which were then abstracted and synthesized into overarching themes. Any discrepancies in the analysis were resolved by returning to the original transcripts for renewed discussion until a consensus was reached. (5) Developing an Exhaustive Description and (6) Extracting the Fundamental Structure: The research team engaged in synthesizing interpretation and discussion based on all generated themes, aiming to form a holistic understanding of the experience. This process culminated in extracting an essential statement that captured the core and universal elements of the experience (i.e., the central finding reported in this study). (7) Verifying the Description: This central finding, along with supporting representative quotations, was returned to five participants for member checking in an accessible language. All participants confirmed the accuracy of the description. Subtle semantic feedback (e.g., perceptions of the word “compromise”) was incorporated into the final revision.

To ensure the rigor and trustworthiness of the research, the following measures were implemented. (1) Investigator Triangulation and Peer Debriefing: The aforementioned coding and theme development process constituted investigator triangulation. Furthermore, a third senior qualitative researcher, who was not involved in the initial coding, was invited to conduct a peer debriefing on the preliminary thematic structure, and their suggestions were integrated into the final analysis. (2) Reflexive Practice: The research team (all with clinical backgrounds) maintained analytical memos to continuously document and critically examine their potential presuppositions. These preconceptions were consciously “bracketed” during team discussions to prioritize fidelity to participants’ narratives. (3) Assessment of Data Saturation: After analyzing the 9th transcript, two consecutive interviews yielded no new meaning units or thematic dimensions. Upon collective deliberation by the research team, thematic saturation was determined to have been reached. Therefore, data from the first 9 participants were included in the final analysis.

## 3 Results

### 3.1 Theme 1: adverse physiological experience

#### 3.1.1 Rash

The skin rash induced by EGFRIs was consistently reported not merely as the most common symptom but also as the primary and most visually salient signature of treatment toxicity. Thus, patients were cast into the role of observers of their own bodily transformation, employing detailed sensory and narrative language to document an unwelcome transformation of their physical selves. As P1 detailed, “It started with just a little redness. For me, it began around my mouth, then slowly spread to the sides of my nose, and finally crept up to my forehead. Later, it showed up on my upper body and back.” Similarly, P9 noted its evolving pattern: “It started as a small patch of redness and slowly spread. After a long time, the color gets a little darker.” This detailed tracking reflects anxious engagement with a symptom that is both visibly expanding and mapping itself onto their identity. Furthermore, patients actively compared its texture and appearance to familiar conditions such as acne, highlighting their cognitive struggle to categorize this new drug-induced reality. P4 emphasized the distinctive tactile quality that led to recognition: “I noticed it felt very granular at first, like tiny little bumps. Initially, I just assumed it was my own acne flaring up, but then it got worse and worse, and that’s when I realized it was definitely related to the medication.”

#### 3.1.2 Pruritus

Pruritus transcends being a mere symptom checklist item for patients on EGFRIs; it emerges as a pervasive and tormenting lived experience that dominates consciousness and erodes quality of life. This experience is characterized not only by intense physical sensations but also by psychological entrapment and a futile struggle for control. P5, who uses metaphorical language to express pruritus: “My primary complaint is the incessant itching; I scratch constantly. I think there is poison gas in my skin; after scratching this skin, poison gas came out. Every day I itch, every day I am uncomfortable.” Beyond the sensation itself, the unpredictable onset and compulsive behavioral response create a cycle of frustration and helplessness, where the patient’s own actions seem to worsen the condition. P2 highlighted the distress of its unpredictable onset: “The itching was initially tolerable but gradually intensified, especially as the rash became more pronounced. The onset of itching is unpredictable, which is extremely frustrating.” P4 detailed the vicious, self-perpetuating cycle this creates: “Sometimes the urge to scratch becomes compulsive, forcing me to scratch the specific site of the itch. I realized the more I scratched, the more intense the urge became, leading to an increased rash. It felt like a vicious circle. The itching is truly tormenting, comparable to constant, unremitting pain if left unmanaged.”

#### 3.1.3 Dryness, desquamation, and fissures

The symptoms of dryness, desquamation, and fissures represent fundamental and persistent alterations in the skin state. P2 articulated this profound shift in bodily constitution, stating, “My skin, including the scalp, feels noticeably rougher. It has become severely dry since I started using this targeted drug. I used to have relatively oily skin, but that constitution has completely changed.” P5 vividly depicted the experience of desquamation, explaining, “This patch of skin here is peeling; it’s extremely dry, like dandruff. I feel like this area is just detaching layer by layer. I feel like my skin is constantly shedding, and even light touching causes dander to fall.” This altered state manifests as a stark, uneven bodily geography, creating a tangible contrast between the rash-affected and unaffected areas. This visible and tactile disparity serves as a constant reminder of the treatment’s localized assault. P9 highlighted this stark contrast, noting, “The difference between my two legs is quite distinct. Clearly, the skin on the left is rougher than the right, especially in the vicinity of the rash, where the texture is significantly coarser.” This condition culminates in seasonal suffering, progressing from dryness to painful fissures that actively hinder daily life. P8 and P6 both described the progression to painful fissures, with P6 employing a powerful metaphor: “Last winter, my hands and feet developed deep cracks, resembling fissures in an old, abandoned wall, which were dry and painful.” P6 further emphasized the seasonal dimension of this suffering, concluding, “This situation is acceptable with tolerance in summer, but winter is really uncomfortable.”

### 3.2 Theme 2: negative and positive emotional experiences coexist

#### 3.2.1 Irritability and distress

Patients with cancer often feel irritable and distressed by skin toxicity symptoms caused by EGFRIs. This emotion permeates their lived experience, primarily manifesting in three interconnected dimensions: assault on self-image, disruption of basic well-being, and a compounded sense of existential injustice. The visible rash compounds the emotional burden of the illness. This is directly expressed by P2: “The increasing severity of the rash makes me feel extremely vexed and frustrated. My mood was already poor due to the cancer diagnosis, and now this rash, especially on my face—it’s so disfiguring!” The Disruption of Fundamental Well-being and Sleep. Uncontrollable symptoms, such as nocturnal itching, invade critical restorative spaces, creating a cycle of exhaustion. P4 provides a detailed account of this intrusion: “I experience intense distress and irritability because of this rash. Nocturnal pruritus frequently causes delayed sleep onset, and I only eventually fall asleep from sheer exhaustion. My greatest fear is being abruptly woken up by a sudden bout of itching in the middle of the night—that is excruciating! (helplessly laughed) Once woken by the itch, it is profoundly difficult to return to sleep, leading to intense annoyance.” The Compounded Sense of Existential Injustice. Patients perceive side effects as an unfair addition to their primary illness, leading to deep-seated frustration. P9 articulates this sentiment: “I sometimes question whether I am the sole person suffering from this misfortune. Others are healthy, yet I developed cancer. Other cancer patients use targeted therapy too, and why skin toxicity symptoms come for me? I occasionally reflect on whether this indicates an unfortunate destiny.”

#### 3.2.2 Worry and fear

Some patients with cancer will feel worried and afraid due to skin toxicity symptoms, worried that skin toxicity symptoms will affect their prognosis and subsequent treatment. These fears are future-oriented, centering on two core threats: the permanence of bodily change and the stability of essential treatment. Fear of Permanent Bodily Transformation. The rapid and visible onset of symptoms triggers anxiety about lasting damage to physical identity. P3’s narrative quickly escalates to this fear: “I only commenced this medication recently, and my skin was normal beforehand. Within a few days, the rash began. I feel the drug reaction is excessively rapid; I find it intolerable. Perhaps I am simply too sensitive (bitterly laughed). I am concerned about whether my skin will recover or if this condition will persist, potentially resulting in disfigurement!” Anxiety about Treatment Compromise and Wasted Investment. These side effects create a dilemma between symptom management and preservation of the efficacy of costly life-sustaining therapies. P9 poignantly describes this conflict: “My skin was not like this initially. I occasionally feel profoundly unfortunate. Targeted drugs are so expensive, and I have invested substantial financial resources, only to encounter various adverse reactions. The physician informed me that if the skin condition is intolerable, dose reduction or drug change might be necessary. I am extremely reluctant to pursue this option. I feel distressed because I am significantly worried about adverse effects on the efficacy of my primary cancer treatment. This severely affects my motivation for treatment (sigh).”

#### 3.2.3 Feel the love from family and friends

During the illness, patients with cancer feel concerned and cared for by their family and friends. This support was vital and was expressed through several key forms of help and connections. Patients received crucial help, both with the economic burden of treatment and in managing daily discomfort. This is seen in P3’s account, which highlights both financial and physical care: “This targeted drug is quite costly, yet my family never hesitated or questioned the financial burden of my treatment, for which I am deeply grateful. When I experience pruritus, my mother patiently rubs and massages the area repeatedly to provide relief. I believe I have the most supportive parents possible.” Sustained companionship countered feelings of isolation. Acts such as being accompanied to appointments or receiving regular check-ins reassured patients that they were not alone or a burden. P5 described the importance of these consistent actions: “My children personally transport me to the hospital for every appointment. My friends frequently call to express concern about my physical and mental health and have not distanced themselves due to my illness.” Illness often leads to a renewed appreciation of relationships. P5 reflected on this change: “I believe that through this illness, I have gained a new perspective or profound insight. I was likely less observant before, but the illness has increased my sensitivity to the love and concern surrounding me.” Illness as a Lens for New Emotional Clarity. The experience of vulnerability can sharpen one’s perception, allowing deeper recognition and appreciation of existing connections that may have been previously overlooked.

#### 3.2.4 Indirect enhancement of anticancer determination

Symptoms of skin toxicity enhance confidence and determination to fight cancer in some patients. This is because patients actively interpret their symptoms within a positive framework, viewing them either as tangible signs of treatment efficacy or as a shared challenge that can be overcome. This positive meaning-making is illustrated by P5, who reframed the rash as direct evidence of the drug’s activity, even interpreting it as a process of “detoxification” linked to encouraging biomarker trends: “The physician informed me that these symptoms are normal following drug initiation, which indicates that the medication is active. I even rationalized that the targeted drug was assisting my body in detoxification, manifested by the onset of the rash. I routinely monitor my tumor markers, and they have shown a decline since starting the drug, which is encouraging. Given that I have only recently started this treatment, I am confident that the ‘internal toxicity’ will gradually diminish, and my overall condition will continue to improve.” P8 described drawing strength from a fellow patient’s story: “I confirmed with my doctor that these are mainly side effects of the targeted drug and that immediate resolution is challenging. I have an acquaintance who underwent the same treatment for the same condition. He experienced similar symptoms but endured them. He advised me to maintain confidence, explaining that his symptoms were initially more severe but eventually resolved even while continuing the medication. He also stated that the drug was highly effective in controlling his tumor. Given its strong therapeutic effect in his case, I am hopeful it will yield similar results for me.” Learning that other patients had successfully endured similar challenges provided tangible hope, normalized their struggles, and strengthened their resolve to continue treatment.

### 3.3 Theme 3: impact on daily life

#### 3.3.1 Decreased social willingness

Some patients with cancer change their image and have reduced social willingness because of skin toxicity symptoms. This social withdrawal stems from a profound fear of negative judgment and damaged self-image, leading to active strategies of avoidance and concealment. The visibility of symptoms triggers anxiety regarding mockery, pity, or social rejection. P3 described this concern in the context of peer interactions: “I have stopped attending school now. Although the rash on my face is mild, it still causes me great concern. I fear that my classmates may assign me derogatory nicknames or view me negatively. This, compounded by the frequent need for hospital visits, has resulted in a marked reduction in social contact.” P9 similarly feared the judgment of other people: “A cancer diagnosis is not something I regard as an honor, and I prefer that others do not know about it. When I return to the village, people often inquire about the rash on my face. I am reluctant to elaborate, fearing they may look down on me or circulate baseless rumors, so I always find a plausible excuse.” To manage the risk of exposure to skin toxicity symptoms, patients physically hid their symptoms as much as possible and withdrew from the social spaces and activities they once enjoyed. P6 detailed this retreat from public life and hobbies: “When my skin is itching, I feel extremely uncomfortable, and I avoid going out to see people. I used to play ping-pong with my friends, but now, with this appearance, I am afraid of alarming others. I am simply unwilling to leave the house, and my outings have become very infrequent; I remain at home unless absolutely necessary. Even when I do go out, I will wear a hat or mask and cover my face. I have suspended my previous outdoor hobbies to prioritize treatment at this stage.” P9 also emphasized the protective behaviors of self-concealment and social withdrawal: “I sometimes try to return home unnoticed, or I wear a hat and mask, completely covering myself before going out, for fear of being recognized. In this way, I can protect myself as much as I can. Typically, I stay at home and avoid unnecessary excursions.”

#### 3.3.2 Decreased drug compliance

Some patients with cancer often take fewer doses or miss doses because of unbearable skin toxicity symptoms and decreased medication adherence. This non-adherence arises from a fundamental conflict: the urgent need to relieve unbearable physical suffering directly clashes with the imperative to maintain potentially life-saving treatment. Patients found themselves trapped in this dilemma, feeling caught between following medical advice and obeying their bodies’ demands for relief. P4 expressed this internal conflict, revealing how the side effects prompted thoughts of abandoning the prescribed regimen: “The side effects of this targeted drug are truly debilitating. I secretly considered requesting a change in my treatment regimen because the discomfort was too severe. However, my doctor advised me that this regimen was currently the optimal choice, necessitating my continued endurance. Consequently, I feel obligated to adhere to the doctor’s instructions.” Faced with this impossible choice, “self-adjustment” of the dose has emerged as a last-resort strategy for regaining a sense of control, even when aware of the potential risks to efficacy. P9’s narrative provides a clear example of this risky compromise, highlighting a resigned acceptance of potentially reduced treatment outcomes: “After recognizing that the symptoms were adverse effects of the targeted drug, I previously attempted to reduce the dosage independently, occasionally taking less medication. My family cautioned against interrupting the drug, but I found the side effects unbearable. The doctor warned me that the dose reduction might compromise the drug’s original efficacy, asking me to prepare myself mentally. I believe I can accept that outcome; since the cancer has already metastasized, I feel that the situation cannot deteriorate significantly further. I am resigned to the situation and feel like I’m leaving the outcome to fate.”

### 3.4 Theme 4: multiple coping styles

#### 3.4.1 Neglect and disregard

Skin toxicity symptoms are mostly insidious at onset, leading patients to initially overlook them. This neglect unfolds into two discernible phases: an initial phase of misattribution to benign causes, followed by a later phase of conscious tolerance when the symptoms are recognized as treatment-related. Patients commonly misinterpret early signs as minor, transient skin issues, delaying the correct association with medication. This cognitive delay was evident in their accounts. P5 described this initial misattribution: “I did not notice it initially, thinking it was just a bee or mosquito bite, but then I realized it was not. I even applied household ointments and insect repellents, which provided no relief whatsoever.” Similarly, P7 detailed a gradual process of realization: “The symptom onset occurred a few days after drug initiation. When I first encountered it, I dismissed it, initially thinking I had slept on it incorrectly or that it was pressure-induced. The rash on my face only drew my attention when it became widely distributed. It began as a small red spot, which I dismissed as common acne, and I only noticed its true nature as it slowly progressed.” Even with an awareness of the drug connection, patients may consciously choose to neglect symptoms, rationalizing them as a non-critical trade-off for anticancer treatment. P8 exemplified this stance of deliberate tolerance: “I have been continuously taking the targeted drug, so the symptoms are not entirely resolved, but fortunately, the aggravation has not been observed. I have gradually started to ignore it, choosing to tolerate the skin discomfort; I reason that it is not life-threatening, and I will simply scratch the itch when necessary.” P3’s statement reflects conditional awareness, where attention is reserved for future severity: “Currently, if the overall skin symptoms remain non-severe, I believe they are acceptable, and I do not allocate substantial attention to them. I will focus my attention if the symptoms intensify later.”

#### 3.4.2 Disgust and resistance

As skin toxicity symptoms worsen, patients not only feel disgust and resistance but also take specific actions to cope. Their actions focus on two main areas: managing how others see them and managing their discomfort with the changed appearance. First, patients cope by carefully concealing their skin from that of others. They worry about being judged; therefore, they attempt to hide their rashes. P6 described this focus as hiding symptoms and fearing others’ opinions: “There is significant concern regarding my current skin condition. Wearing clothes provides some concealment, but I worry about others noticing the red or hyperpigmented rashes on my hands and forming negative judgments. Sometimes I feel profoundly anxious, fearing what people might assume about the state of my skin. I intensely dislike these rashes and wish they would simply vanish.” Second, patients cope by avoiding situations that increase their personal distress. This means changing their habits to avoid seeing rashes or exposing them, even in private. P9 explained how she avoids looking at her skin and changes what she wears to feel less troubled: “The skin condition sometimes feels repulsive and unsightly. Seeing my patchy skin in the mirror after bathing makes it incredibly difficult to accept, but thankfully, wearing clothes helps cover it. I previously wore short skirts, but now I am limited to wearing long skirts, solely to prevent the rash from being visible. And I do not like looking in the mirror because I’m afraid of seeing the skin rash on my body.”

#### 3.4.3 Acceptance and compromise

Over time, patients with cancer gradually accept and compromise their skin toxicity symptoms. When coping with persistent symptoms, patients develop distinct strategies for coexistence. One coping strategy is “conditional endurance.” Patients weigh skin symptoms against the critical goal of anticancer treatment. As long as the symptoms do not worsen and core treatment remains effective, patients choose to endure them. P3’s statement exemplifies this pragmatic trade-off: “I believe my symptoms remain mild, possibly because I have only recently started the medication. Beyond the rash, the slight pruritus and dryness are tolerable. Skin issues are minor; as long as my tumor remains controlled, I can endure these symptoms, provided they do not progressively worsen. For me, the current problem is not substantial.” Another coping strategy is “compromise through reframing and social comparison.” By interacting with others, patients assign new, positive meanings to the symptoms (e.g., “a sign that the drug is working”), thereby shifting their perspective from resistance to calm acceptance. P8’s experience clearly illustrates this process of cognitive shift: “When my skin condition was severe, I felt like I was the most unfortunate person alive. Dealing with cancer, and now this appearance, was devastating. However, I later encountered patients in the hospital with similar conditions who offered encouragement, asserting that the rash was a favorable sign, proving the drug’s efficacy, and justifying the financial cost. This allowed me to relax. Subsequently, I gradually reached a state of compromise and equanimity; the rash is not a major obstacle, and the symptoms will eventually improve. Given the current advancements in medical technology, there will always be a solution for my disease.”

#### 3.4.4 Admission and growth

Some patients with cancer transitioned to an active coping phase, characterized by rigorous self-monitoring, experimentation with home remedies, and the development of personalized self-management strategies. P2 described a heightened awareness of physical triggers and a learned behavioral adjustment: “I am slowly discovering certain methods to improve my skin comfort. I recognize my skin is quite delicate; the bath water cannot be too hot, as excessive heat is intolerable—slightly cooler water feels better, while hotter water increases the discomfort. I feel significantly better after showering. Sometimes the itching is so intense that I will take a quick shower specifically for relief.” Building on this attentiveness to daily routines, P3 expanded the scope of self-management to encompass holistic lifestyle and psychological adjustments: “I realized that the facial cleanser I use is quite stimulating to the skin, and I thought that increasing exercise, sun exposure, and sweating might be beneficial. I drink more water, as there seems to be no other immediate intervention. I believe it is best to allow the body to adjust internally while continuing the medication. I am actively trying to maintain a cool physical temperature and a serene psychological state.” Advancing to a more experimental approach, P7 demonstrated how direct trial and evaluation led to the refinement of her care methods: “I prepared self-made iodophor swabs for application, which I found quite soothing. I had previously used alcohol, but some excoriated areas were extremely painful; the alcohol caused a burning sensation and worsened the condition in several spots. For dryness, I previously attempted to moisturize my skin using hand cream or a general body lotion.” Collectively, these narratives illustrate a progressive journey from heightened self-awareness to deliberate adoption and refinement of personalized care practices, marking a significant transition from passive endurance to active self-management and self-growth.

### 3.5 Theme 5: desire for medical support and help

Patients with cancer lack awareness of skin toxicity symptoms and are eager to receive professional medical support. This profound desire reflects patients navigating the uncertainty of self-management, actively seeking authoritative knowledge, and reliable continuity of care to bridge the gap between hospital treatment and lived experience. Faced with unfamiliar symptoms, patients’ primary need is to understand the “what” and “why” and to obtain concrete steps for relief. P1 articulated the need for basic explanation and intervention: “I possess limited health literacy and knowledge, making it difficult for me to understand these medical conditions. Therefore, I make sure to discuss my skin symptoms during the doctor’s ward round, with the hope of receiving interventions to improve my skin condition.” Patients strategically view skin toxicity management as essential for ensuring that their overarching cancer therapy is not interrupted or delayed. P6 emphasized this priority of safeguarding the treatment plan: “I wish to fully restore my skin to its original state. It is also essential to identify the root cause and control the symptoms, as I have scheduled future treatment sessions, and I must not allow the skin problems to delay my overall progress. I urgently require assistance from the physicians and nurses.” Patients anticipate the challenges of managing symptoms at home, expressing a need for guidance that extends past the discharge period, and mitigating fears of being left alone with a worsening condition. P9 expressed anxiety about post-discharge care and the need for ongoing professional access: “This symptom is highly uncomfortable. I am unaware of whether there is a specialized clinic for managing this specific toxicity. Since my family resides in a different region, I worry about the rash worsening after discharge. It would be ideal to have professional doctors and nurses available to provide continuous guidance.”

## 4 Discussion

### 4.1 Improving evaluation and management of skin toxicity

The findings revealed that patients with cancer frequently experience adverse physiological symptoms, such as rash, itching, dryness, desquamation, and fissures, following the initiation of EGFRIs. Therefore, there is a clear recommendation for healthcare professionals to enhance symptom evaluation and management protocols for this patient population. Most skin toxicity symptoms are significantly associated with the dose of EGFRIs (Zhu et al., 2018). Symptoms of early skin toxicity often have an insidious onset and are easily misidentified as common dermatological conditions. Patients tend to disregard or selectively neglect early symptoms such as mild pruritus, dryness, or desquamation, which can inadvertently lead to the progression of skin toxicity and potentially compromise the continuation of the core anticancer regimen in severe cases. Consistent with previous reports, rash was confirmed as the most common and earliest cutaneous toxicity of EGFRIs (Passaro et al., 2014). Dry skin and pruritus are often accompanied by an acneiform rash or occur later, typically within 1–2 months after treatment with EGFRIs (Wu and Lacouture, 2018; Clabbers et al., 2016). Dry skin increases the risk of skin damage and fissures, representing a key factor in the potential for secondary bacterial and viral skin infections. The patients’ limited health literacy regarding EGFRI-induced skin toxicities directly contributes to reduced social engagement, decreased medication adherence, and compromised quality of life. Clinical medical staff must conduct timely and accurate assessments of the severity of skin toxicity, rash density, signs of infection, and subjective experiences of adverse reactions. Concurrently, patients require guidance on implementing appropriate supportive care measures to effectively mitigate the progression of skin toxicity, reduce patient discomfort, and enhance their quality of life.

### 4.2 Provide psychological support and reduce psychological burden on patients

The emergence of EGFRI-induced skin toxicities frequently precipitates negative emotional experiences, including irritability, distress, worry, and fear. Therefore, it is essential for medical staff to provide psychological support to alleviate this psychological burden. Patients receiving targeted therapy already cope with malignant diseases. The addition of visible adverse drug reactions, particularly dense facial rashes and intense pruritus, significantly impacts their body image and mental state, often leading to irritability, anxiety about prognosis, and substantial psychological distress (Ma, 2020). Chronic negative emotional states can disrupt internal physiological homeostasis, potentially inducing psychosomatic disorders that perpetuate and worsen negative affect, thereby creating a vicious cycle (Zhang, 2019). A qualitative study (Lei et al., 2018) on targeted lung patients with cancer highlighted a significant perceived lack of scientific guidance post-discharge, resulting in feelings of helplessness and psychological pressure. This underscores the need for clinical practitioners to prioritize the psychological needs of these patients by offering emotional support and specialized assistance. Skin integrity is fundamentally linked to the maintenance of body image and self-esteem. Persistent and severe dermatological adverse reactions are closely associated with restricted daily activities and deterioration of intimate and interpersonal relationships and can even contribute to communication impairments and social isolation (Yagasaki et al., 2018). Healthcare providers can learn from positive psychological interventions such as focused solving models (Luo et al., 2007), mindfulness therapy (Xiong and Yu, 2011), cognitive behavioral therapy (Thoma et al., 2015), narrative therapy (Sun et al., 2022), and empathy therapy (Neumann et al., 2009) to proactively address and mitigate negative emotions and provide psychological support to cancer patients.

### 4.3 Provide professional guidance and help patients respond effectively

The study identified a progression in coping strategies used by patients with cancer in response to skin toxicity symptoms, including initial neglect and disregard, followed by feelings of disgust and resistance, eventually moving toward acceptance and compromise, and finally achieving acceptance and growth. It is recommended that medical staff identify their coping styles and guide their active coping. Coping style is defined as the cognitive and behavioral approach an individual employs when confronting frustration and stress (Zhang et al., 2005). Due to the insidious and mild nature of EGFRI-induced skin toxicities, patients often exhibited neglect and disregard. However, as symptoms worsened, the rash became persistent, the associated risks were unavoidable, and the impact on body image and daily life was pronounced. Patients frequently demonstrated aversion and resistance. With prolonged illness duration and recurrent symptoms, patients gradually moved toward acceptance or compromise regarding skin toxicities. Through self-adjustment, some patients achieved full acceptance and experienced personal self-growth. A patient’s level of psychological flexibility is a critical factor, as higher flexibility facilitates a more positive attitude toward coping with the disease and its associated symptoms. Therefore, healthcare providers should deliver specialized professional services and guidance, reinforce open communication, encourage patients to disclose and verbalize their skin toxicity experiences, facilitate the stimulation of positive emotional reframing, and ultimately assist patients with cancer in effectively responding to these challenging symptoms.

### 4.4 Improve support systems and promote self-growth

Following the onset of EGFRI-induced skin toxicities, patients reported feeling profoundly supported by their families, which fulfilled their emotional needs, fostered positive emotional experiences, and indirectly fortified their resolve to fight cancer. Based on this finding, improvement of support systems and facilitation of patient self-growth are strongly recommended. Social support systems are crucial for mitigating psychological stress, dismantling psychological barriers, and enhancing the mental health of cancer patients. Research by Liu (2022) indicated that social support and psychological flexibility exert a chain-mediating effect on the quality of life of cancer patients, suggesting that augmenting the degree of social support serves as a viable intervention point for reducing psychological distress. We suggest that medical staff fully leverage external resources, including family and community networks, to enhance patients’ cognitive coping skills and help them maintain a positive emotional state. Simultaneously, it is important to guide relatives and friends to adopt a rational and objective perspective on the patient’s identity, avoiding both overprotection and social alienation, thereby maximizing the mental health benefits of social support. This study revealed that some patients used skin toxicity to achieve self-growth, learn to appreciate their relationships with their parents and children, master life management skills, engage in active exercise, and maintain an optimistic outlook. During the process of managing skin toxicities, medical staff should help patients recognize their potential for self-growth, encourage them to confront life difficulties bravely, create supportive environments to fully capitalize on their inherent strengths, and actively guide them toward positive affect and adaptive behaviors.

### 4.5 Limitations and prospects

The fundamental rationale for including patients receiving both EGFR monoclonal antibodies and tyrosine kinase inhibitors in this qualitative study is rooted in their shared mechanisms of action. Despite the differences in molecular size and binding sites, both drug classes are designed to inhibit the epidermal growth factor receptor (EGFR) signaling pathway. It is this common inhibition of a pathway critical for skin barrier function and follicular integrity that leads to a well-documented, highly overlapping spectrum of dermatological toxicities (Jiang et al., 2022)^.^ Therefore, exploring the common lived experiences of patients facing class-effect toxicity was both methodologically sound and the primary aim of our study. At the same time, while this study elucidates common experiences, any subtle differences in symptom presentation or perception specific to each drug class represent an important focus for future research to optimize individualized care. We suggest that future work explore and compare the potential distinctions in symptom experiences between these two subclasses to further personalize supportive care.

## 5 Conclusion

This qualitative study illuminates the profound impact of EGFRI-induced skin toxicity, revealing an experience that extends beyond physical symptoms to disrupt patients’ psychological well-being, daily life, and self-image. Our findings advocate for a comprehensive patient-centered support model in clinical practice. This model necessitates proactive symptom assessment to identify often-neglected early signs. It also requires integrated psychological support to address significant emotional distress and body image concerns arising from visible symptoms. Furthermore, care must include dynamic, stage-matched guidance that aligns with the patient’s evolving coping journey—from initial neglect and resistance toward eventual acceptance and adaptive self-growth. Finally, clinicians and families should consciously foster supportive environments and reinforce adaptive coping strategies. In summary, addressing EGFRI-induced skin toxicity requires a comprehensive approach that concurrently targets symptom control, psychological distress, adaptive coping, and environmental support.

## Data Availability

The raw data supporting the conclusions of this article will be made available by the authors, without undue reservation.
